# Sleep and BMI in South African urban and rural, high and low-income preschool children

**DOI:** 10.1186/s12889-021-10591-5

**Published:** 2021-03-23

**Authors:** Dale E. Rae, Simone A. Tomaz, Rachel A. Jones, Trina Hinkley, Rhian Twine, Kathleen Kahn, Shane A. Norris, Catherine E. Draper

**Affiliations:** 1 Health through Physical Activity, Lifestyle and Sport Research Centre & Division of Exercise Science and Sports Medicine, Department of Human Biology, Faculty of Health Sciences, University of Cape Town, Cape Town, South Africa; 2 Early Start, Faculty of the Arts Social Science and Humanities, University of Wollongong, Wollongong, Australia; 3 Institute for Physical Activity and Nutrition (IPAN), School of Exercise and Nutrition Sciences, Deakin University, Geelong, Australia; 4 MRC/Wits Rural Public Health and Health Transitions Research Unit (Agincourt), School of Public Health, Faculty of Health Sciences, University of the Witwatersrand, Johannesburg, South Africa; 5 Umeå Centre for Global Health Research, Umeå University, Umeå, Sweden; 6 INDEPTH Network, Accra, Ghana; 7 SAMRC/Wits Developmental Pathways for Health Research Unit, School of Clinical Medicine, Faculty of Health Sciences, University of the Witwatersrand, Johannesburg, South Africa

**Keywords:** Sleep, Adiposity, Early childhood, Low- and middle-income country

## Abstract

**Background:**

The extent to which income setting or rural and urban environments modify the association between sleep and obesity in young children is unclear. The aims of this cross-sectional observational study were to (i) describe and compare sleep in South African preschool children from rural low-income (RL), urban low-income (UL) and urban high-income (UH) settings; and (ii) test for associations between sleep parameters and body mass index (BMI).

**Methods:**

Participants were preschoolers (5.2 ± 0.7y, 49.5% boys) from RL (*n* = 111), UL (*n* = 65) and UH (*n* = 22) settings. Height and weight were measured. Sleep, sedentary behaviour and physical activity were assessed using accelerometery.

**Results:**

UL children had higher BMI z-scores (median: 0.39; interquartile range: − 0.27, 0.99) than the UH (− 0.38; − 0.88, 0.11) and RL (− 0.08; − 0.83, 0.53) children (*p* = 0.001). The UL children had later bedtimes (*p* < 0.001) and wake-up times (*p* < 0.001) and shorter 24 h (*p* < 0.001) and nocturnal (*p* < 0.001) sleep durations than the RL and UH children. After adjusting for age, sex, setting, SB and PA, for every hour less sleep obtained (24 h and nocturnal), children were 2.28 (95% CI: 1.28–4.35) and 2.22 (95% CI: 1.27–3.85) more likely, respectively, to belong to a higher BMI z-score quartile.

**Conclusions:**

Shorter sleep is associated with a higher BMI z-score in South African preschoolers, despite high levels of PA, with UL children appearing to be particularly vulnerable.

**Supplementary Information:**

The online version contains supplementary material available at 10.1186/s12889-021-10591-5.

## Background

Insufficient or short sleep has been associated with obesity and increased cardiometabolic risk in adults [1–3] and children [4, 5]. Duration of sleep in children has been shown to vary between countries, ethnic groups and income settings. The International Study of Childhood Obesity, Lifestyle and the Environment affirms that sleep duration and timing in children aged 9–11 years differs between countries and that associations between sleep parameters and activity or sedentary parameters were stronger in the high- compared to low-income countries [6]. Within high-income countries, sleep duration and patterns vary between children from different ethnic groups, with non-white children or minority groups being more likely to have shorter sleep [7] or later bedtimes [8]. There is also evidence to suggest that children from higher income families have longer sleep [9], earlier bedtimes [8] and better sleep efficiency [10] than those from lower income settings.

South Africa (SA) is an upper middle-income country characterised by large discrepancies in socioeconomic status (SES) with nearly half of the population (49.2%) living in poverty [11] in both rural and urban settings. It is undergoing an epidemiological transition; obesity is on the rise across all age groups [12] and non-communicable diseases are now responsible for more deaths annually than infectious diseases [13].

Preschool-aged children warrant investigation since early childhood is a period understood to predict obesity in adolescence [14] and 13% of preschool children in SA are overweight or obese [12]. For 3 to 5-year-old children, 10-13 h of sleep is recommended per 24 h period (comprising nocturnal sleep and daytime naps) to promote optimal health and development [15–18]. Only one study has been published on objectively measured sleep in SA preschool children. This study observed short nocturnal sleep (9.3 ± 0.8 h) in preschool-aged children in an urban, low-income setting in SA, where even after the inclusion of daytime naps, 24 h sleep duration was less than 10 h in 38% of the children [19]. Whether or not this observation of shorter sleep is also present in the rural setting or is a consequence of income status has not been established in this age group. The aims of this study were to (i) describe and compare sleep in preschool-aged South African children from rural low-income, urban low-income and urban high-income settings and (ii) test for associations between sleep and body mass index.

## Methods

This cross-sectional, observational study is a secondary analysis of a previously published, separate study, investigating body composition, physical activity and gross motor skills in urban and rural preschoolers [20]. Only relevant methodological components are described in more detail below. The present study includes 198 of the original 268 participants for whom valid sleep datasets were available. Compliance within the urban high-income was very poor, resulting in a small group size.

### Setting

Data were collected from children (5.19 ± 0.69 years) living in rural low-income (RL, *n* = 111), urban low-income (UL, *n* = 65) and urban high-income (UH = n=22) settings. Data collection took place between July and September 2012 (UH and UL), and between June and August 2014 (RL). While a range of geographical settings was included, this sample was not nationally representative. The RL site was a rural village in north-east SA characterised by high unemployment [21], and households with limited access to electricity and running water [22], and a high dependence on social grants. Access to this site was facilitated through the SAMRC/Wits Rural Public Health and Health Transitions Research Unit (Agincourt). Both urban settings were in south-west SA. The UL setting was a suburb comprising a mix of informal and low-cost housing in which common challenges are unemployment, overcrowding and crime. The UH setting comprised adjacent suburbs where the population density is approximately 15 times lower than the UL setting. The area has high-quality private and public schools, private health facilities and services, public parks and green spaces, expensive retailers and well-serviced amenities.

### Participants and recruitment

UH and UL preschools were selected using convenience sampling and were intentionally economically diverse, taking into account geographical location and community-level factors indicating socioeconomic circumstances, such as those mentioned above. Recruitment of the RL preschool was coordinated through the Public Engagement Office of the SAMRC/Wits-Agincourt Unit. All parents were provided with written information about the study, as well as with instructions (in their home language) for the use of accelerometers. Where requested, parent meetings were arranged to assist with recruitment. All children between the ages of 3 and 5 years who were: attending preschool; willing and able to take part in the testing for this study; and for whom written informed parental consent was obtained, were included in this study.

Ethical approval was obtained from the University of Cape Town Human Research Ethics Committee (HREC Ref. No.: 237/2012), the University of the Witwatersrand Human Research Ethics Committee (Medical) (M140250), and the Mpumalanga Provincial Departments of Health and Education.

### Detailed procedures

#### Anthropometry

Height (m) and weight (kg) were measured to calculate body mass index (BMI, kg/m^2^). Height and weight measurements were conducted with the children’s shoes and heavy clothing removed. A portable stadiometer (Leicester 214 Transportable Stadiometer; Seca GmbH & Co, Hamburg, Germany) was used to measure height to the nearest millimetre. To measure body weight, the children stood centred on a calibrated scale (Soehnle 7840 Mediscale Digital; Soehnle Industrial Solutions GmbH, Backnang, Germany). Height and weight measurements were done by a trained member of the research team.

Age standardised scores for BMI (BMI z-score), height (height-for-age z-score, HAZ) and weight (weight-for-age z-score WAZ) were computed using the World Health Organisation AnthroPlus software (http://www.who.int/growthref/tools/en/). The International Obesity Task Force cut-offs were used to classify children based on the following BMI categories: thinness, normal weight, overweight, obese and morbidly obese [23]. For the regression analyses, since the BMI z-score data were not normally distributed, BMI z-score quartile groups were used: quartile 1: *n* = 50, median: − 1.21 (interquartile range: − 1.48, − 0.88); quartile 2: *n* = 49, − 0.28 (− 0.49, − 0.11); quartile 3: *n* = 50, 0.24 (0.13, 0.45); quartile 4: *n* = 49, 1.13 (0.82, 1.48).

#### Sleep, sedentary behaviour and physical activity

Actigraph GT3X+ accelerometers (Actigraph LLC, Pensacola, FL; USA) were used for the objective measurement of sleep, sedentary behaviour (SB) and physical activity (PA) over a 7-day period as previously described [19]. For a valid sleep dataset, a child needed to have at least 3 valid sleep nights (> 160 min), including at least 1 weekend night. Sunday to Thursday nights were taken to represent week nights, Friday and Saturday nights weekend nights, Monday to Friday mornings week mornings and Saturday and Sunday mornings weekend mornings. In the absence of sleep diaries or an accepted sleep-wake algorithm for hip- or waist-worn ActiGraph devices in preschool-aged children, visual inspection [24] was used to obtain nocturnal sleep variables (bedtime, hh:mm; wake-up time, hh:mm; time-in-bed, h), and daytime nap duration (h) using ActiLife version 6 (ActiLife software; Pensacola, FL; USA). Compliance with sleep guidelines for this age group [15–18] was determined using 24 h sleep duration (nocturnal time-in-bed plus daytime nap duration) and children were classified as meeting sleep guidelines if their average 24 h sleep duration was between 10 and 13 h.

Datasets for SB and PA were deemed valid if they had 7 h of valid wear-time (excluding sleep time) on 3 weekdays and 1 weekend day [25]. Non-wear time was defined as 20 min of consecutive zeroes and was removed from the data [26]. Cut-off points chosen to identify SB and moderate- to vigorous-intensity physical activity (MVPA) in this study were < 25 counts/15 s [27] and > 420 counts/15 s [28], respectively. Duration (min) of time spent in SB, light physical activity (LPA, > 25 and < 420 counts/15 s), MVPA and total PA (light- to vigorous-intensity physical activity, LMVPA) were calculated. Children were classified as meeting South African [18] and World Health Organisation [17] PA guidelines for this age group if they spent an average of ≥180 min/day in LMVPA, inclusive of 60 min/day of ‘energetic play’ (operationalised as MVPA).

### Data and statistical analyses

Data are presented as mean ± standard deviation, median (interquartile range) or percentage. The Shapiro-Wilks test was used to assess normality of data. Between-group analyses were conducted using an independent t-test, Mann-Whitney U test, one-way analysis of variance (ANOVA) or Kruskal-Wallis tests. Post hoc analyses were conducted using Bonferroni or Mann-Whitney U tests. Distributions were compared using Pearson’s Chi-squared and correlations determined using Pearson’s r or Spearman’s rho tests. Ordered logistic regression analyses were used to evaluate associations between sleep and BMI. The dependent variable was BMI z-score quartile group and the independent variables were 24 h sleep duration and nocturnal time-in-bed. Regression analyses were adjusted for age, sex, setting, SB and MVPA a priori since these variables have known associations with sleep and BMI. Data were analysed using Stata (v13, STATA Corp, College Station, TX) and significance accepted for *p* < 0.05.

## Results

### Descriptive characteristics

Descriptive characteristics of the participants stratified by sex and setting are presented in Table 1. The proportion of boys (*n* = 98) and girls (*n* = 100) in the study was similar (*p* = 0.841). The boys were older (*p* = 0.037), had higher BMI (*p* = 0.009) and BMI z-scores (*p* = 0.017) and accumulated more MVPA each day (*p* < 0.001) than the girls. Post hoc analyses indicate that the RL children were younger than the UL (*p* < 0.001) children, although all were preschool-aged. The UL children had higher BMI (*p* = 0.001) and BMI z-scores (*p* = 0.017) than the RL (BMI: *p* = 0.001, BMI z-score: *p* = 0.001) and UH (BMI: *p* = 0.002, BMI z-score: *p* = 0.002) children. More UL children were classified as overweight/obese compared to the RL and UH groups (*p* = 0.012) and fewer UL children were classified as thin compared to the other two groups (*p* = 0.016). The UH children had significantly higher HAZ scores than the UL (*p* = 0.008) and RL (*p* = 0.010) children. The RL children engaged in less sedentary behavior than the UL (*p* < 0.001) and UH (*p* = 0.006) children, while the UH children accumulate less LPA and LMVPA compared to the UL (LPA: *p* < 0.001, LMVPA: *p* < 0.001) and RL (LPA: *p* < 0.001, LMVPA: *p* < 0.001) children. The majority of this cohort met the PA guidelines [17, 29] for this age group (98%), and this distribution did not differ between the three settings (RL: 98.2%, UL: 98.5%, UH: 95.5%; Chi^2^ = 0.812, *p* = 0.666).

**Table 1:** Descriptive characteristics of participants stratified by sex and group

	All (***n*** = 198)	Boys (***n*** = 98)	Girls (***n*** = 100)	***P***-value	Rural Low (***n*** = 111)	Urban Low (***n*** = 65)	Urban High (***n*** = 22)	***P***-value
Age (y)	5.19 ± 0.69	5.29 ± 0.70	5.09 ± 0.67	***0.037***	5.05 ± 0.64^a^	5.36 ± 0.71^a^	5.38 ± 0.74	***0.006***
BMI (kg/m^2^)	15.4 (14.3, 16.2)	15.5 (14.9, 16.2)	14.9 (14.1, 16.1)	***0.009***	15.1 (14.1, 16.0)	15.8 (14.9, 16.7)^b^	14.8 (14.3, 15.5)	***0.001***
BMI-z score	0.05 (−0.72, 0.68)	0.16 (− 0.34, 0.73)	− 0.23 (− 0.85, 0.53)	***0.017***	−0.08 (− 0.83, 0.53)	0.39 (− 0.27, 0.99)^b^	−0.38 (− 0.88, 0.11)	***0.001***
HAZ	−0.31 ± 1.01	−0.34 ± 0.98	−0.27 ± 1.04	0.658	−0.36 ± 0.97	−0.42 ± 1.03^b^	0.32 ± 0.99	***0.007***
WAZ	− 0.17 ± 1.02	−0.09 ± 0.90	−0.25 ± 1.13	0.271	−0.30 ± 0.96	−0.02 ± 1.15	−0.02 ± 0.87	0.167
Thinness (%)	20.7	14.0	27.0	0.062	26.1	10.8^b^	22.7	
Normal (%)	71.2	77.0	64.0		68.5	73.8	77.3	***0.017***
Overweight/obese (%)	8.1	7.0	9.0		5.4	15.4^b^	0	
SB (min/d)	308.8 (279.3, 340.3)	308.2 (272.8, 337.3)	310.0 (281.6, 341.7)	0.509	292.1 (263.8, 330.6)^b^	321.1 (298.2, 345.7)	321.3 (304.5, 347.8)	***< 0.001***
LPA (min/d)	337.8 ± 37.2	334.3 ± 37.3	341.3 ± 39.7	0.072	343.6 ± 35.6	339.1 ± 37.1	304.9 ± 29.0^b^	***< 0.001***
MVPA (min/d)	123.6 (102.4, 146.9)	138.2 (110.3, 155.4)	112.6 (91.9, 134.4)	***< 0.001***	121.8 (97.5, 146.9)	132.6 (112.8, 154.0)	112.0 (94.3, 132.0)	0.125
LMVPA (min/d)	464.1 ± 56.3	471.5 ± 56.9	456.8 ± 55.1	0.065	469.9 ± 54.8	469.8 ± 55.2	417.5 ± 46.9^b^	***< 0.001***

Data are presented as mean ± SD, median with interquartile range or percentage

Significance was determined using independent t-tests, Mann Whitney-U, one-way ANOVA, Kruskal-Wallis ANOVA or Chi^2^ analyses

Significance was accepted at *p* < 0.05

*BMI* Body mass index, *HAZ* Height-for-age z-score, *WAZ* Weight-for-age z-score, *SB* Sedentary behaviour, *LPA* Light-intensity physical activity, *MVPA* Moderate- to vigorous-intensity physical activity, *LMVPA* Light- to vigorous-intensity physical activity

^a^Post hoc analyses indicate significant differences between two marked groups

^b^Post hoc analyses indicate that marked group is significantly different to the other two groups

### Sleep characteristics

Average, week and weekend sleep characteristics of the children are presented in Table 2. Boys napped less during the day on average (*p* = 0.008) and on weekends (*p* = 0.002) and had later bedtimes during the week (*p* = 0.049) than girls. Post hoc analyses indicate that the UL children had later bedtimes than the RL (average: *p* < 0.001, week: *p* < 0.001 and weekend: *p* < 0.001) and UH (average: *p* < 0.001, week: *p* < 0.001 and weekend: *p* < 0.001) children; later wake-up times than the RL (average: *p* < 0.001, week: *p* < 0.001 and weekend *p*<:0.001) and UH (average: *p* = 0.001, week: *p* = 0.027 and weekend: *p* < 0.001) children; and shorter nocturnal time-in-bed durations than the RL (average: *p* < 0.001, week: p < 0.001, weekend: *p* < 0.001) and UH (average: *p* < 0.001, week: *p* < 0.001, weekend: *p* < 0.001) children. The RL children had significantly longer weekend nocturnal time-in-bed durations than the UH (*p* < 0.001) children. Daytime naps were identified in 55% (*n* = 109) of the children in this sample. There were no differences in daytime nap duration between the three groups, but more napping took place during the week (94%) than on weekends (20%, *p* < 0.001).

**Table 2:** Sleep characteristics of participants stratified by sex and setting group

	All (***n*** = 198)	Boys (***n*** = 98)	Girls (***n*** = 100)	***P***-value	Rural Low (***n*** = 111)	Urban Low (***n*** = 65)	Urban High (***n*** = 22)	***P***-value
**Average**								
Bedtime (hh:mm)	20:08 (19:32, 21:02)	20:12 (19:38, 21:12)	20:01 (19:22, 21:00)	0.085	19:38 (19:11, 20:08)	21:20 (21:01, 21:41)^c^	19:56 (19:35, 20:22)	***< 0.001***
Wake-up time (hh:mm)	06:40 (06:22, 07:09)	06:42 (06:22, 07:18)	06:39 (06:22, 07:00)	0.242	06:26 (06:11, 06:44)	07:11 (06:53, 07:32)^c^	06:43 (06:21, 07:19)	***< 0.001***
Nocturnal time-in-bed (h)	10.48 ± 0.77	10.44 ± 0.07	10.52 ± 0.08	0.478	10.76 ± 0.68	9.92 ± 0.68^c^	10.76 ± 0.61	***< 0.001***
Nap duration (h)^a^	0.97 (0.80, 1.24)	0.86 (0.77, 1.13)	1.07 (0.90, 1.41)	***0.008***	0.94 (0.77, 1.22)	1.03 (0.83, 1.33)	0.89 (0.81, 1.02)	0.367
**Week**								
Bedtime (hh:mm)	20:02 (19:29, 21:01)	20:13 (19:37, 21:11)	19:54 (19:19, 20:53)	***0.049***	19:40 (19:10, 20:06)	21:18 (20:59, 21:41)^c^	19:49 (19:29, 20:13)	***< 0.001***
Wake-up time (hh:mm)	06:34 (06:11, 06:59)	06:37 (06:11, 07.11)	06:31 (06:11, 06:52)	0.268	06:17 (06:04, 06:34)	07:04 (06:40, 07:24)^c^	06:44 (06:23, 07:05)	***< 0.001***
Nocturnal time-in-bed (h)	10.39 ± 0.80	10.33 ± 0.07	10.45 ± 0.09	0.310	10.63 ± 0.68	9.81 ± 0.70^c^	10.90 ± 0.70	***< 0.001***
Nap duration (h)^a^	1.00 (0.80, 1.30)	0.86 (0.77, 1.12)	1.11 (0.91, 1.48)	***0.002***	1.01 (0.79, 1.31)	1.03 (0.85, 1.45)	0.83 (0.74, 0.95)	0.182
**Weekend**								
Bedtime (hh:mm)	20:13 (19:30, 21:11)	20:19 (19:33, 21:14)	20:05 (19:26, 21:02)	0.350	20:21 (19:58, 20:49)	21:31 (20:55, 22:03)^c^	19:35 (19:13, 20:13)	***< 0.001***
Wake-up time (hh:mm)	06:59 (06:31, 07:32)	07:04 (06:30, 07:39)	06:58 (06:32, 07:23)	0.276	06.37 (06:17, 07:21)	07:41 (07:05, 07:59)^c^	06:46 (06:20, 07:10)	***< 0.001***
Nocturnal time-in-bed (h)	10.72 (10.04, 11.41)	10.57 (10.02, 11.44)	10.81 (10.05, 11.37)	0.995	10.32 (10.04, 10.88)^b^	10.10 (9.63, 10.58)^c^	11.14 (10.52, 11.54)^b^	***< 0.001***
Nap duration (h)^a^	0.90 ± 0.32	0.83 ± 0.34	0.99 ± 0.30	0.132	0.89 ± 0.30	0.85 ± 0.37	0.95 ± 0.30	0.644

Data are presented as mean ± SD or median with interquartile range

Significance was determined using independent t-tests, Mann Whitney-U, one-way ANOVA or Kruskal-Wallis ANOVA tests

^a^Sample size for naps: Average: *n* = 103, weekdays: *n* = 103 and weekend days: *n* = 40

^b^Post hoc analyses indicate significant differences between two marked groups

^c^Post hoc analyses indicate that marked group is significantly different to the other two groups. Significance was accepted at *p* < 0.05

**Fig. 1:**
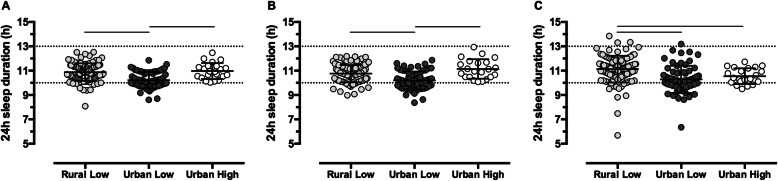
Average (**a**), week (**b**) and weekend (**c**) 24 h sleep durations for the RL (*n* = 111), UL (*n* = 65) and UH (*n* = 22) groups. Individual data points and mean ± SD are presented. Dotted lines at 10 h and 13 h represent the recommended sleep duration range for preschoolers. Solid lines indicate post hoc differences between groups following a one-way ANOVA. RL: Rural low-income group; UL: Urban low-income group, UH: Urban high-income group

Figure 1 illustrates 24 h sleep durations of children in the RL, UL and UH groups on average (A), during the week (B) and on weekends (C). The UL group had shorter 24 h sleep durations compared to the RL (average: *p* < 0.001 and week: *p* < 0.001) and UH (average: *p* < 0.001 and week: *p* < 0.001) groups. On weekends, the RL children had longer 24 h sleep durations than the UH (*p* < 0.001) and UL (*p* < 0.001) children. Of the entire cohort, 80.8% met current sleep guidelines [15–18]. Fewer children in the UL group (55%) met the guidelines compared to those in the RL (92%) and UH (100%) groups (*p* < 0.001). Removing daytime naps (i.e. evaluating nocturnal time-in-bed) reduced compliance in all three groups to 87% (RL), 42% (UL) and 91% (UH).

### Correlation analyses

Within-group correlations between average 24 h sleep duration and nocturnal time-in-bed with BMI z-score are presented in Additional Figure A1. No significant relationships were observed, although there was a trend for 24 h sleep duration to be negatively correlated with nocturnal time-in-bed in the UL group (*n* = 65, *r* = − 0.241, *p* = 0.053, Additional Figure A1B). No within-group correlations were observed between average bedtime, wake-up time and BMI z-score (Additional Figure A2). In all three groups, average nocturnal time-in-bed was negatively correlated with average bedtime and positively correlated with average wake-up time (Additional Figure A3).

### Regression analyses

**Fig. 2:**
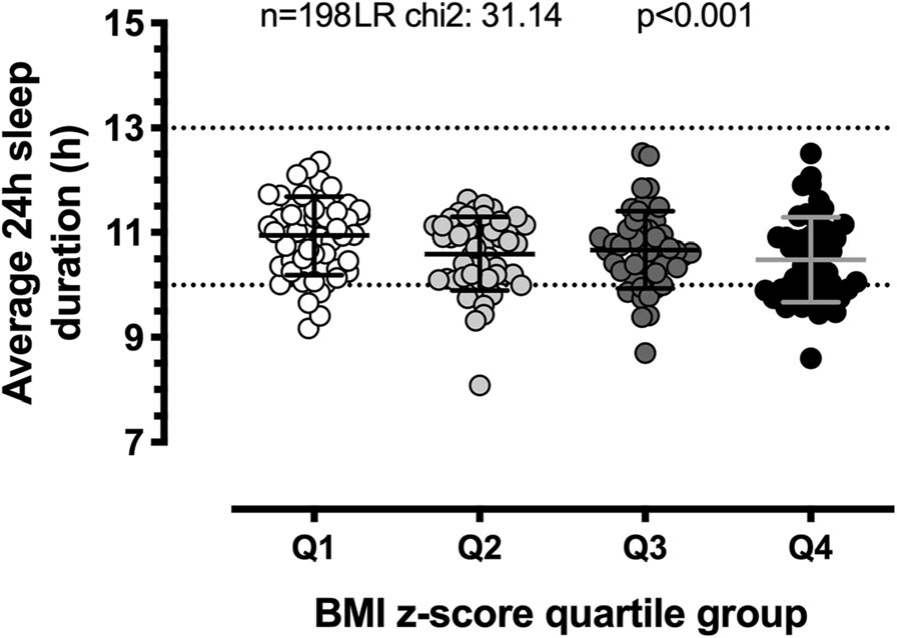
Association between 24 h sleep duration and BMI z-score quartiles. Individual data points and mean ± SD are presented. Dotted lines at 10 h and 13 h represent the recommended sleep duration range for preschoolers. Q1: lowest BMI z-score quartile group, Q4: highest quartile group. Analyses were performed using ordered logistic regression, adjusting for sex, age, group, sedentary behaviour and LMVPA

The association between average 24 h sleep duration and BMI z-score quartiles for all participants is displayed in Fig. 2. There was a linear relationship between the two variables with the lowest BMI z-score quartile comprising longer sleeping children and the highest quartile comprising shorter-sleeping children (*p* < 0.001). Table 3 summarises the ordered logistic regression results for BMI-z quartile (dependent variable) and average 24 h sleep duration (independent variable, model 1) and average nocturnal time-in-bed (independent variable, model 2). For every hour less sleep obtained in a 24 h period, children were 2.38 times more likely to belong to a higher BMI z-quartile (95% confidence interval (CI): 1.28, 4.35, *p* = 0.006) when adjusting for sex, age, setting, sedentary behaviour and LMVPA. Additionally, UL children were 2.91 times more likely to fall into a higher BMI z-quartile than UH children (95% CI: 1.15, 7.40; *p* = 0.025). For every hour less nocturnal sleep obtained, children were 2.22 times more likely to fall into a higher BMI z-quartile (95% CI: 1.27, 3.85; *p* = 0.004). Regression analyses were also run with average bedtime (model 3), average wake-up time (model 4) and average nap duration (model 5) as independent variables and BMI-z quartile (dependent variable), but no associations were observed (Additional Table A1).

**Table 3:** Ordered logistic regression models associating BMI-z quartile (dependent variable) and average 24-h sleep duration (Model 1) and nocturnal time-in-bed (Model 2)

Model 1						Model 2					
			OR	95% CI	***p***-value				OR	95% CI	***p***-value
**Ave 24-h sleep duration**			0.42	0.23, 0.78	0.006	**Ave nocturnal time-in-bed**			0.45	0.26, 0.79	0.005
**Covariates**	*Sex: Boy v girl*		0.61	0.36, 1.03	0.066	**Covariates**	*Sex: Boy v girl*		0.57	0.33, 0.97	**0.037**
	*Age*		0.78	0.51, 1.20	0.261		*Age*		0.72	0.46, 1.13	0.153
	*Group:*	*UL v UH*	2.91	1.15, 7.40	**0.025**		*Group:*	*UL v UH*	2.48	0.96, 6.38	0.060
		*RL v UH*	1.14	0.48, 2.69	0.773			*RL v UH*	1.15	0.49 2.73	0.747
	*Sedentary Behaviour*		0.99	0.98, 1.00	**0.009**		*Sedentary Behaviour*		0.99	0.98, 1.00	**0.018**
	*LMVPA*		0.99	0.98, 1.00	0.128		*LMVPA*		1.00	0.99, 1.00	0.254
**Model**: *n* = 198, LR chi2 = 31.14, *p* < 0.001						**Model**: *n* = 198, LR chi2=31.62, *p* < 0.001					

*OR* Odds ratio, *CI* Confidence interval, *UL* Urban low-income group, *UH* Urban high-income group, *RL* Rural low-income group, *LMVPA* Low- to vigorous-intensity physical activity

## Discussion

This is the first study to compare sleep (objectively measured or parent-report) in preschool children from a range of income settings in South Africa, and to investigate the relationship between sleep and obesity in these groups. This study found that for every hour less 24 h or nocturnal sleep obtained, these preschool children were more than twice as likely to have a higher BMI, after adjusting for age, sex, setting, sedentary behaviour and physical activity. This is similar to what has been reported in two other studies in preschoolers from other countries [30, 31] and highlights the need to address sleep duration as a risk factor for obesity in South African children as young as 3–5 years in age. This is particularly relevant in South Africa where 13% of children under 5 years are overweight or obese [12]. Although not significant, the trend for average 24 h sleep duration to be negatively correlated with BMI z-score in the UL children suggests that the urban low-income setting is particularly vulnerable. Just over half of the children in the UL group (55%) met the sleep guidelines (i.e. 10–13 h in a 24 h period) [15–18] compared to most of those in the RL (92%) and UH (100%) groups. Despite high levels of physical activity, children from the UL setting had higher BMI z-scores, slept less in a 24 h period and at night, and had later bedtimes and wake-up times than children from the RL and UH settings.

The shorter sleep observed in the low- compared to high-income children within the urban setting is consistent with what has been reported in children [32, 33] and adults [34] in other countries. Low-income settings are typically characterised by an adverse social environment (i.e. high disorder and low safety and social cohesion), which in turn has been associated with shorter [35], worse quality [36] sleep in adults. Furthermore, in low-income homes, housing type, crowding, number of sleeping rooms and people per bed may influence sleep duration and quality. For example, the association between lower SES and poorer subjective ratings of sleep and higher daytime sleepiness in 11-year-old children was mediated by a higher exposure to poor sleeping conditions (noisy, light, uncomfortable, too hot or cold) [37]. Additionally, 1-year-olds from families of lower SES and poor home environment quality are more likely to have shorter, less consistent sleep timing at the age of 8 years [38].

Within low-income settings, we observed shorter sleep in the urban (9.90 ± 0.68 h) compared to rural (10.76 ± 0.68 h) environment. Parent-reported sleep of a group of preschoolers comprising both urban and rural American Indian preschool has been described (10.15 ± 0.97 h), but urban and rural children were not compared [31]. Similar to our results, but in slightly older children (6–10 year-olds), shorter sleep duration was reported in Thai children living in the city compared to rural areas [39]. No difference in self-reported sleep duration, however, was observed between urban and rural 12-year-old Ugandan children [40]. One possible explanation for the RL children obtaining longer sleep in the present study might relate to their being fewer environmental barriers to good sleep (noise, light, crowding, safety) in a rural compared to urban home, despite other disadvantages of low SES.

Nocturnal time-in-bed was driven by both later bedtimes and wake-up times in all three groups, and later wake-up times observed in the UL children were unable to offset the shortened sleep opportunity caused by later bedtimes. This raises two important points. First, later bedtimes might reflect limited sleep health knowledge amongst the parents or caregivers of this group and, as such, less enforcement of an earlier bedtime or bedtime routine. Indeed it has been shown that preschool children in lower SES homes are less likely to have regular bedtime routines [8]. A longitudinal study observed that 5–9 year olds with no bedtime routine or age-appropriate bedtimes were more likely to have higher BMIs in adolescence [41]. Second, the later sleep timing (both later bedtime and wake-up time) seen in the UL group may entrain a later circadian rhythm in these children. Not only would this make it more difficult to obtain longer sleep when earlier wake-up times are required for school or extramural activities, later sleep timing has also been associated with less healthy food choices (fewer vegetables and heathy proteins and more processed or fried foods) in preschool children in the USA [42].

Daytime napping rescued 24 h sleep duration to some extent in the UL group. This has also been observed in another SA study of low-income preschool children from a different urban low-income setting [19]. The concern is that while napping may well be typical at preschools, once children enter the primary school phase, daytime napping usually falls away. If these UL children are unable to lengthen their nocturnal sleep in the absence of daytime napping, they may be at risk for chronic sleep deprivation in their crucial foundation years of school.

Finally, there has been a recent trend to observe movement patterns encompassing physical activity, sedentary behaviour, screen time and sleep over a 24 h period in relation to health [17, 43, 44]. The findings from this study compliment the sleep message of the new SA 24 h movement guidelines [18] in which parents, caregivers and teachers at early childhood development facilities are encouraged to help their children attain the 10-13 h of sleep recommended for preschool children.

A strength of this study is that sleep was measured objectively and we are among the first to describe and compare sleep in an economically diverse sample of young children. One limitation relates to light exposure, since the urban and rural sites were located in different parts of the country with varying day lengths. We do not believe this to have had a major effect on sleep timing in the present study since the day length difference between the two sites was only 30 min. We also acknowledge that since our data were collected during the winter months (June to August), the results may be open to seasonality influences. Again, however, we estimate this effect to be minimal since seasonal difference between daylight hours in summer and winter (10-14 h) and even daytime temperatures are moderate. Thus the expected impact on sleep timing would certainly be less compared to countries at more extreme latitudes. A further limitation relates to the convenience sampling method used, which means that our data are not necessarily generalizable or representative of South Africa, and that there is a risk for selection bias. We also acknowledge the small sample size in the urban high-income group, for whom compliance with wearing the accelerometer at night significantly reduced the availability of valid sleep datasets in this group. Finally, future studies exploring the relationship between movement behaviours (physical activity, sedentary behaviour), sleep and obesity would benefit from including a dietary component. The use of a randomised sampling technique to ensure representation of the context in question should also be considered in future studies.

## Conclusions

Our findings suggest that nearly half of the preschool aged children in this study living in an urban low-income setting in South Africa may not be obtaining sufficient sleep and, despite high levels of physical activity, may be at risk for weight gain. Given the importance of maintaining a healthy weight in this age group to protect them from obesity in adolescence, they may well benefit from interventions designed to promote healthy sleep. These might include education of parents and caregivers regarding the importance of sleep for the physical, emotional and cognitive well-being of preschool-aged children, as well as guidelines regarding age-appropriate sleep durations and bedtime routines. While these data relate to South African preschoolers conveniently sampled from three small but diverse communities, they may be applicable to any country with social disparity or with existing rural communities.

## Supplementary Information

**Additional file 1: Figure A1.** Correlations between average 24h sleep duration (A, B, C), average nocturnal time-in-bed (D, E, F) and BMI z-score for the three groups. BMI z-score: body mass index age-standardised score. Analyses were performed using Pearson’s correlation test.

**Additional file 2: Figure A2.** Correlations between average bedtime (A, B, C), average wake-up time (D, E, F) and BMI z-score. BMI z-score: body mass index age-standardised score. Analyses were performed using Pearson’s correlation or Spearman’s rho tests.

**Additional file 3: Figure A3.** Correlations between average nocturnal time-in-bed and average bedtime (A, B, C) with average wake-up time (D, E, F). Analyses were performed using Pearson’s or Spearman’s correlation tests.

**Additional file 4: Table A1.** Ordered logistic regression models associating BMI z-score quartile (dependent variable) with average bedtime (Model 3), wake-up time (Model 4) and nap duration (Model 5).

## Data Availability

The datasets used and/or analysed during the current study are available from the corresponding author on reasonable request.

## Abbreviations

ANOVA: Analysis of variance
BMI: Body mass index
BMI z-score: Body mass index-for-age score
CI: Confidence interval
HAZ: Height-for-age score
PA: Physical activity
LPA: Light-intensity physical activity
LMVPA: Light- to vigorous-intensity physical activity
MVPA: Moderate- to vigorous-intensity physical activity
OR: Odds ratio
SA: South Africa
SB: Sedentary behaviour
SD: Standard deviation
SES: Socioeconomic status
RL: Rural low-income
UH: Urban high-income
UL: Urban low-income
WAZ: Weight-for-age score
