# Supplementary figures and images for: Sleep and BMI in South African urban and rural, high and low-income preschool children
Source: BMC Public Health. 2021 Mar 23;21:571. doi: 10.1186/s12889-021-10591-5 (PMC7986550; doi:10.1186/s12889-021-10591-5)

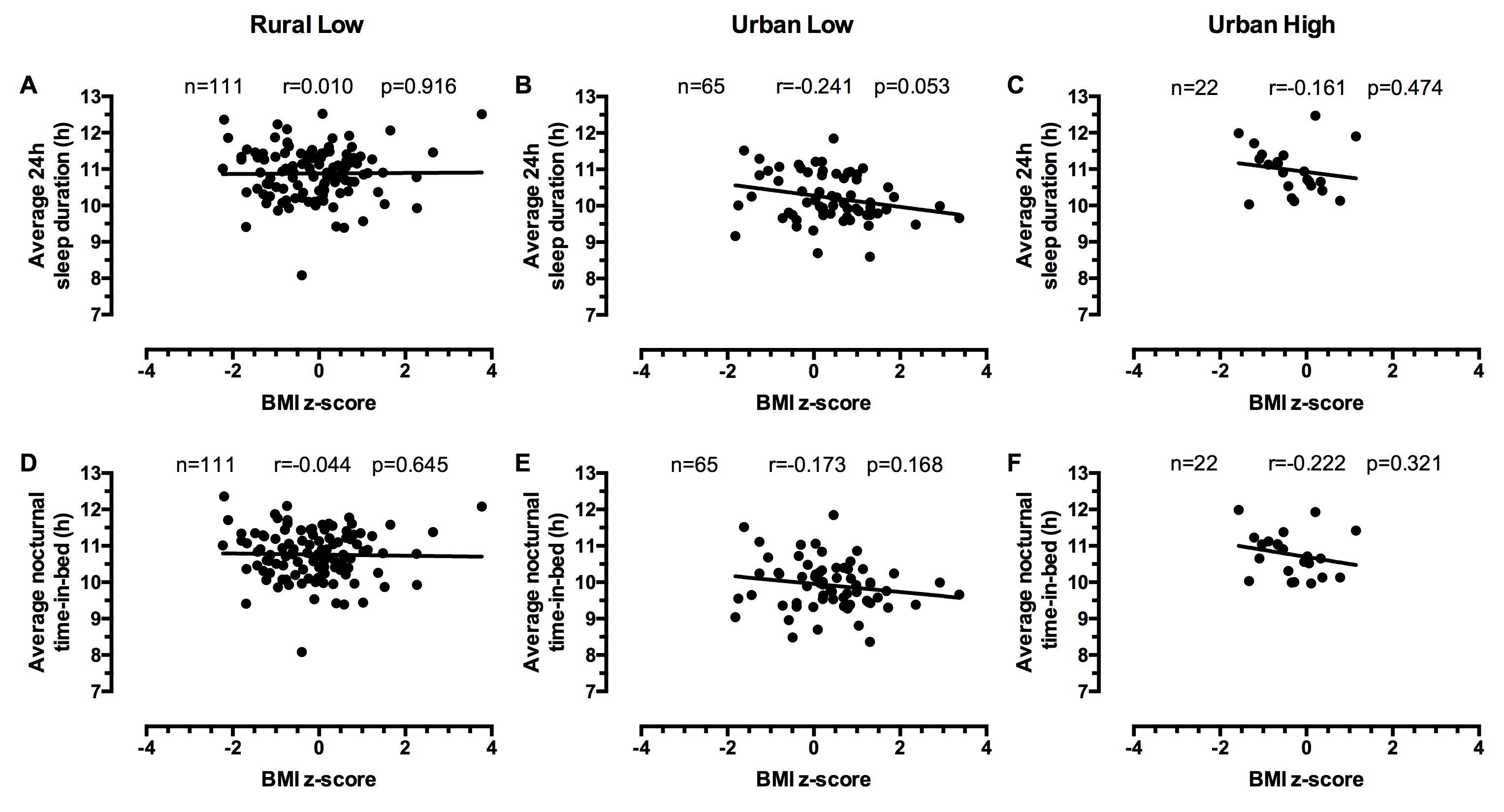

Supplement: Supplementary file 1 — Additional file 1: Figure A1. Correlations between average 24h sleep duration (A, B, C), average nocturnal time-in-bed (D, E, F) and BMI z-score for the three groups. BMI z-score: body mass index age-standardised score. Analyses were performed using Pearson’s correlation test. [file 12889_2021_10591_MOESM1_ESM.tiff]

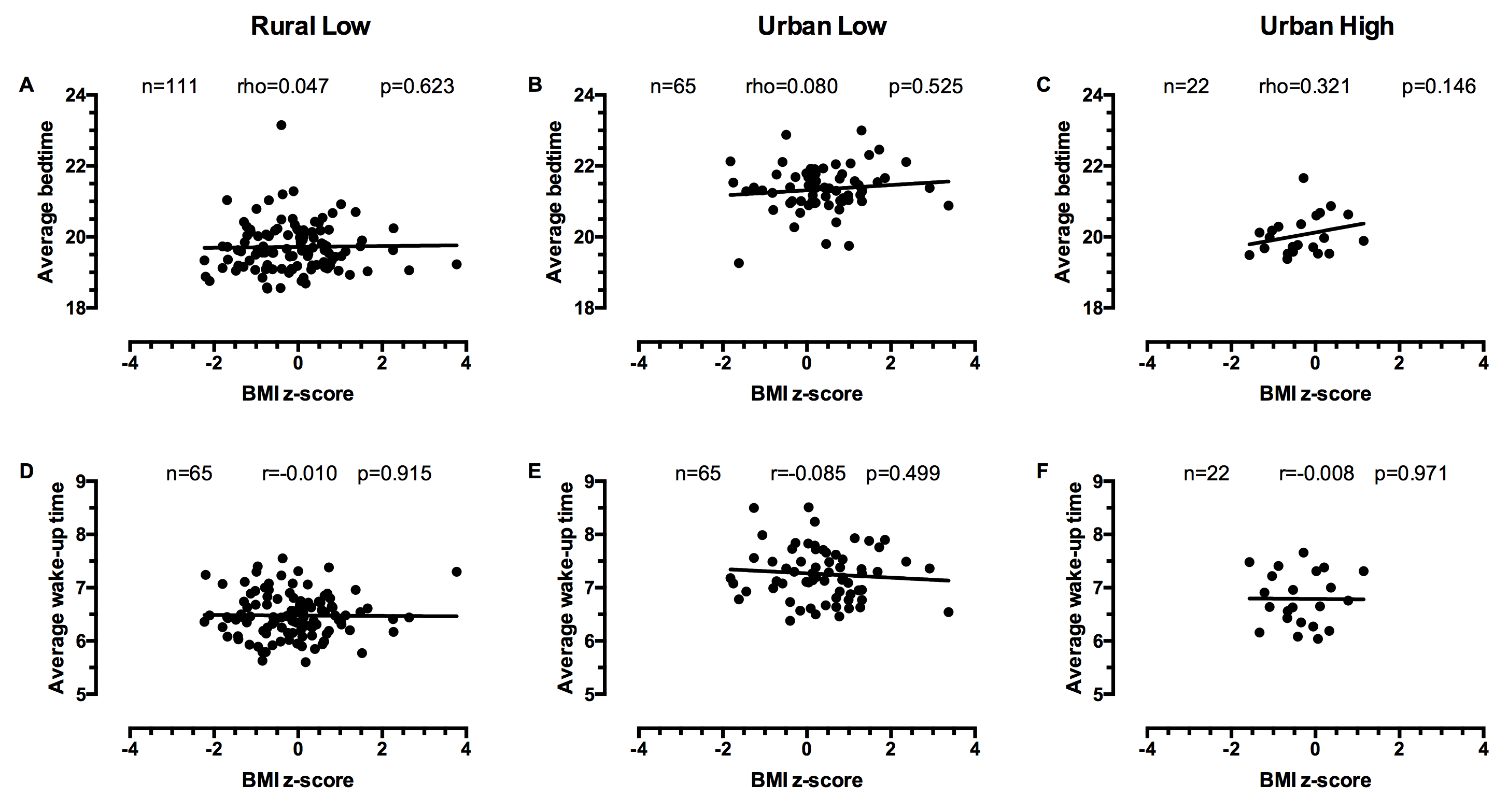

Supplement: Supplementary file 2 — Additional file 2: Figure A2. Correlations between average bedtime (A, B, C), average wake-up time (D, E, F) and BMI z-score. BMI z-score: body mass index age-standardised score. Analyses were performed using Pearson’s correlation or Spearman’s rho tests. [file 12889_2021_10591_MOESM2_ESM.tiff]

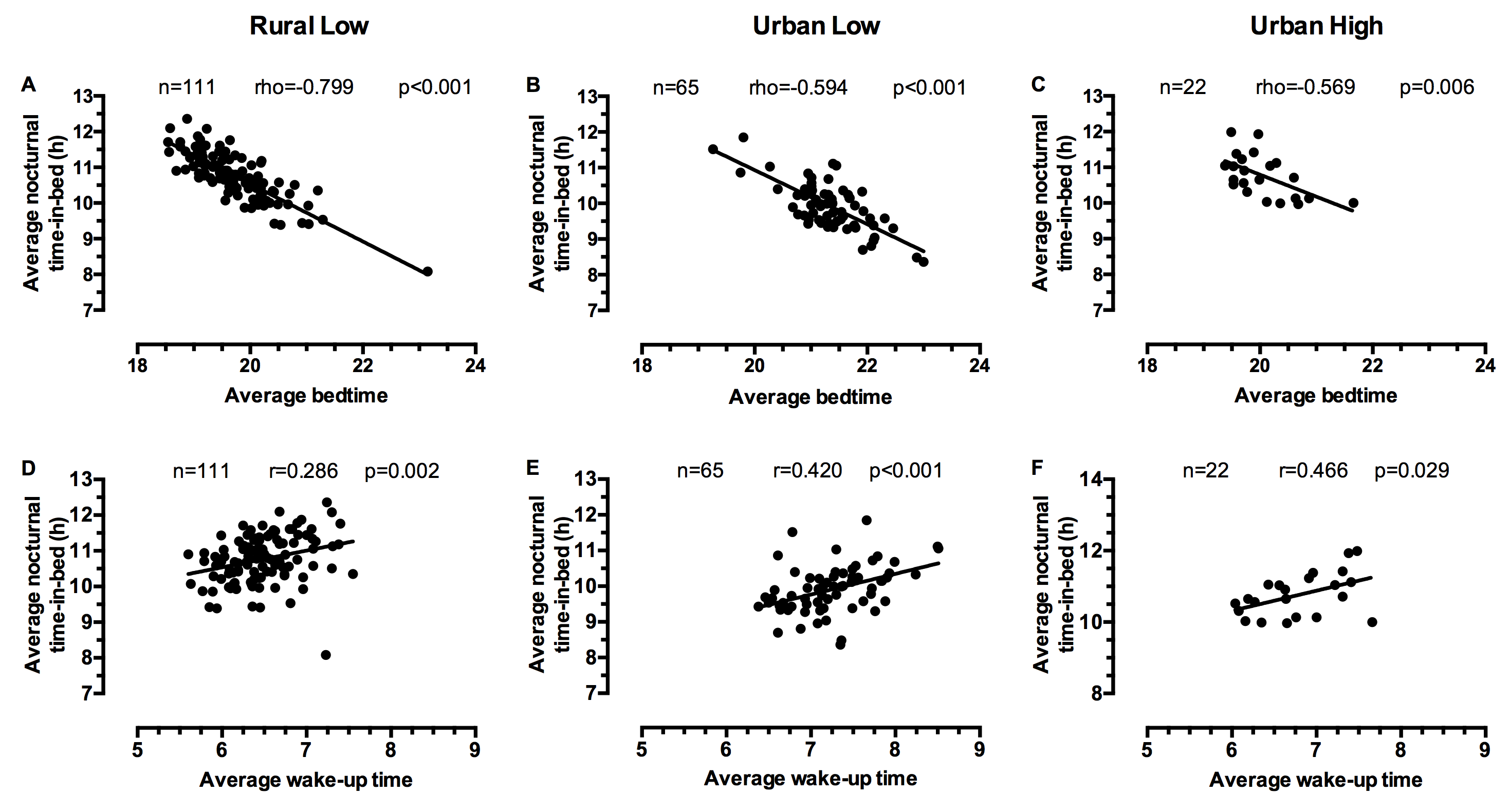

Supplement: Supplementary file 3 — Additional file 3: Figure A3. Correlations between average nocturnal time-in-bed and average bedtime (A, B, C) with average wake-up time (D, E, F). Analyses were performed using Pearson’s or Spearman’s correlation tests. [file 12889_2021_10591_MOESM3_ESM.tiff]
