# Supplementary material for: Sleep and BMI in South African urban and rural, high and low-income preschool children
Source: BMC Public Health. 2021 Mar 23;21:571. doi: 10.1186/s12889-021-10591-5 (PMC7986550; doi:10.1186/s12889-021-10591-5)
Supplement: Supplementary file 4 — Additional file 4: Table A1. Ordered logistic regression models associating BMI z-score quartile (dependent variable) with average bedtime (Model 3), wake-up time (Model 4) and nap duration (Model 5). [file 12889_2021_10591_MOESM4_ESM.docx]

**Additional Table A1. Ordered logistic regression models associating BMI z-score quartile (dependent variable) with average bedtime (Model 3), wake-up time (Model 4) and nap duration (Model 5).**

| **Model 3** | | | | | | | |  | **Model 4** | | | | | | |  | **Model 5** | | | | | | | | | | |  |
| --- | --- | --- | --- | --- | --- | --- | --- | --- | --- | --- | --- | --- | --- | --- | --- | --- | --- | --- | --- | --- | --- | --- | --- | --- | --- | --- | --- | --- |
|  | | **OR** | | **95% CI** | | **p-value** | |  |  | | | **OR** | | **95% CI** | **p-value** |  |  | | **OR** | | | **95% CI** | | | | **p-value** | | |
| **Ave bedtime** | | 1.40 | | 0.88, 2.22 | | 0.152 | |  | **Ave wake-up time** | | | 0.69 | | 0.39, 1.23 | 0.209 |  | **Ave nap duration** | | 1.51 | | | | 0.49, 4.61 | | 0.470 | | |  |
| **Covariates** | *Sex: Boy v girl* | | 0.59 | | 0.35, 1.01 | | 0.052 |  | **Covariates** | *Sex: Boy v girl* | 0.58 | | 0.34, 0.98 | | 0.042 |  | **Covariates** | *Sex: Boy v girl* | | 0.68 | 0.33, 1.41 | | | 0.301 | | |  |  |
|  | *Age* | | 0.80 | | 0.51, 1.24 | | 0.311 |  |  | *Age* | 0.87 | | 0.57, 1.34 | | 0.537 |  |  | *Age* | | 1.01 | 0.56, 1.80 | | | 0.975 | | |  |  |
|  | *Group: UL v UH* | | 2.33 | | 0.83, 6.55 | | 0.110 |  |  | *Group: UL v UH* | 4.15 | | 1.51, 11.41 | | 0.006 |  |  | *Group: UL v UH* | | 2.16 | 0.68, 6.82 | | | 0.189 | | |  |  |
|  | *RL v UH* | | 1.20 | | 0.50 2.86 | | 0.681 |  |  | *RL v UH* | 1.02 | | 0.43, 2.44 | | 0.964 |  |  | *RL v UH* | | 1.43 | 0.48, 4.25 | | | 0.514 | | |  |  |
|  | *Sedentary Behaviour* | | 0.99 | | 0.99, 1.00 | | 0.148 |  |  | *Sedentary Behaviour* | 1.00 | | 0.99, 1.00 | | 0.264 |  |  | *Sedentary Behaviour* | | 1.00 | 0.99, 1.01 | | | 0.903 | | |  |  |
|  | *LMVPA* | | 1.00 | | 0.99, 1.01 | | 0.976 |  |  | *LMVPA* | 1.00 | | 0.99, 1.01 | | 0.732 |  |  | *LMVPA* | | 1.01 | 1.00, 1.01 | | | 0.274 | | |  |  |
| **Model**: n=198, LR chi^2^=25.63, p<0.001 | | | | | | | |  | **Model** n=198, LR chi^2^=24.13, p<0.001 | | | | | | |  | **Model**: n-198, LR chi^2^=7.92, p=0.340 | | | | | | | | | | | |

BMI z-score: body mass index age-standardised score, OR: Odds ratio, CI: confidence interval, UL: urban low-income group, UH: urban high-income group, RL: rural low-income group, LMVPA: low, moderate and vigorous intensity physical activity. Significance was accepted for p<0.050.
